# Robot and mechanical testing of a specialist manual toothbrush for cleaning efficacy and improved force control

**DOI:** 10.1186/s12903-022-02211-4

**Published:** 2022-06-08

**Authors:** Amina Acherkouk, Marco Götze, Andreas Kiesow, Anantha Ramakrishnan, Sandra Sarembe, Tomas Lang, Peter Gaengler

**Affiliations:** 1 GSK Medical Affairs, Weybridge, UK; 2 Fraunhofer Institute for Microstructure of Materials and Systems IMWS, Halle, Germany; 3 Institute for Oral Medicine, University of Witten, Herdecke, Germany

**Keywords:** Toothbrushing, Manual toothbrush, Toothbrush design, Interproximal surface cleaning, Plaque removal, Cleaning efficacy, Robot simulation, Brushing simulator, Typodont

## Abstract

**Background:**

Toothbrushes require flexibility to access all dental surfaces and remove plaque effectively, but they should also aim to prevent or limit overbrushing and consequent damage to teeth and gums. In two studies, the physical properties and cleaning performance of specialist test toothbrushes with flexible necks were compared to a reference rigid-necked toothbrush.

**Methods:**

In Study 1, a universal testing machine (Instron E 10,000) with a specially designed setup was used to test the deflection behaviour of toothbrush head and neck. Untufted toothbrushes were fixed in a custom holder and force was applied to the head while the deflection was measured. In Study 2, one control and five test toothbrushes were assessed using a robot system to simulate the cleaning of artificial plaque from defined surfaces of artificial replicated human teeth in a model oral cavity (typodonts).

**Results:**

Study 1 showed that the flexible-neck toothbrush deflected 2 to 2.5 times more than the rigid-neck reference toothbrush when same force was applied to the toothbrush head. Study 2 revealed that all five test toothbrushes showed statistically superior simulated plaque removal to the reference toothbrush. This superiority was observed for all test toothbrushes employing horizontal and rotating brushing action (all *p* = 0.001) but only three of the five toothbrushes when vertical brushing was employed (all *p* = 0.001). Cleaning efficacy of the test toothbrushes was demonstrated both interdentally and at the gumline locations. The Complete Protection toothbrush showed the most effective cleaning performance followed by the Repair and Protect and Rapid Relief toothbrushes.

**Conclusion:**

The addition of a flexible-neck component to the toothbrush designs helped to reduce stiffness and may allow more effective cleaning compared to rigid designs with controlled force distribution on the teeth and gums. This may help to provide plaque control at all potential risk areas in an in vitro robot model and could support good oral hygiene in-use.

**Supplementary Information:**

The online version contains supplementary material available at 10.1186/s12903-022-02211-4.

## Background

The routine removal of plaque from exposed tooth surfaces using manual toothbrushes remains a critical component of oral health. This is emphasised by infrequent brushing being associated with increased incidence of dental caries and periodontal disease [1–3]. Toothbrushes should provide flexibility, allowing the brush fibres to reach all the accessible dental surfaces. Toothbrushes must also allow enough force to be applied for effective plaque removal but importantly, they should also prevent overbrushing [4–7]. Such use of improper or excessive force during toothbrushing is a known risk factor for developing cervical abrasion lesions, as well as gingival recession, which in turn can cause dentine hypersensitivity [8, 9]. 

Clinical studies are commonly used to evaluate how new toothbrushes perform in the hands of test subjects, however, variations in patients’ oral health, brushing technique, applied force, and compliance with instructions, along with other inconsistencies, can result in data that are not entirely comparable and consequentially limit their reliability [10, 11]. However, robot-based brushing models can play a key role in assessing toothbrush potential due to their ability to reproducibly apply the consistent force and cleaning action to a set of typodonts coated with uniform quantities of ‘artificial’ plaque for each test run [12, 13].

During the past 25 years, increasingly sophisticated versions of these robot systems have been developed to more accurately compare the performance of toothbrushes in vitro, and have provided valuable information allowing objective comparisons between designs [14–16]. These systems have been used to assess plaque removal and determine the effects of brush wear and dentifrice use.

This article reports data from two studies that assessed experimental toothbrush designs (GSK Consumer Health), which incorporates a flexible element in their necks, designed to look like a ball joint, between the toothbrush head and handle. Manual toothbrush users typically employ brushing forces of approximately 1.5–3.25 N, [17–21] and previous studies have shown that higher brushing force directly correlates with higher abrasive dentine wear [22, 23]. These experimental product designs aim to change the angle of the brushing head towards the inbetween regions of buccal and lingual/palatinal tooth surfaces, and to reduce the brushing force that is transmitted to the tooth and gum surfaces. This feature improves the biophysical brushing action and mitigates the risks for tooth wear, receding gums, and dentine hypersensitivity while also delivering efficacious cleaning. These flexible-neck toothbrush designs were compared to a classic rigid-neck design, hereafter referred to as the ‘control’ toothbrush. To evaluate the contact force and deflection behaviour of the flexible- and rigid-neck toothbrushes, they were mechanically tested without bristles in the first study. To assess the cleaning performance of this flexible-neck design, five variants, differentiated by their distinct bristle configurations (hereafter collectively referred to as ‘test’ toothbrushes) were assessed using a robot tooth-cleaning evaluation system in the second study. These test toothbrushes were assessed in comparison to a control toothbrush with a ‘standard’ flat-trim bristle configuration. 

The purpose of these current studies was, firstly, to mechanically evaluate the test and control toothbrushes to compare the levels of deflection as a function of the force applied. The second of the two studies specifically aimed to compare the cleaning efficacy of five test versus control toothbrushes using a robot test of cleaning efficacy by plaque planimetry and at brushing forces of 2.5 newtons [N]. 

## Methods

### Study 1—Mechanical testing of toothbrush deflection

**Fig. 1:**
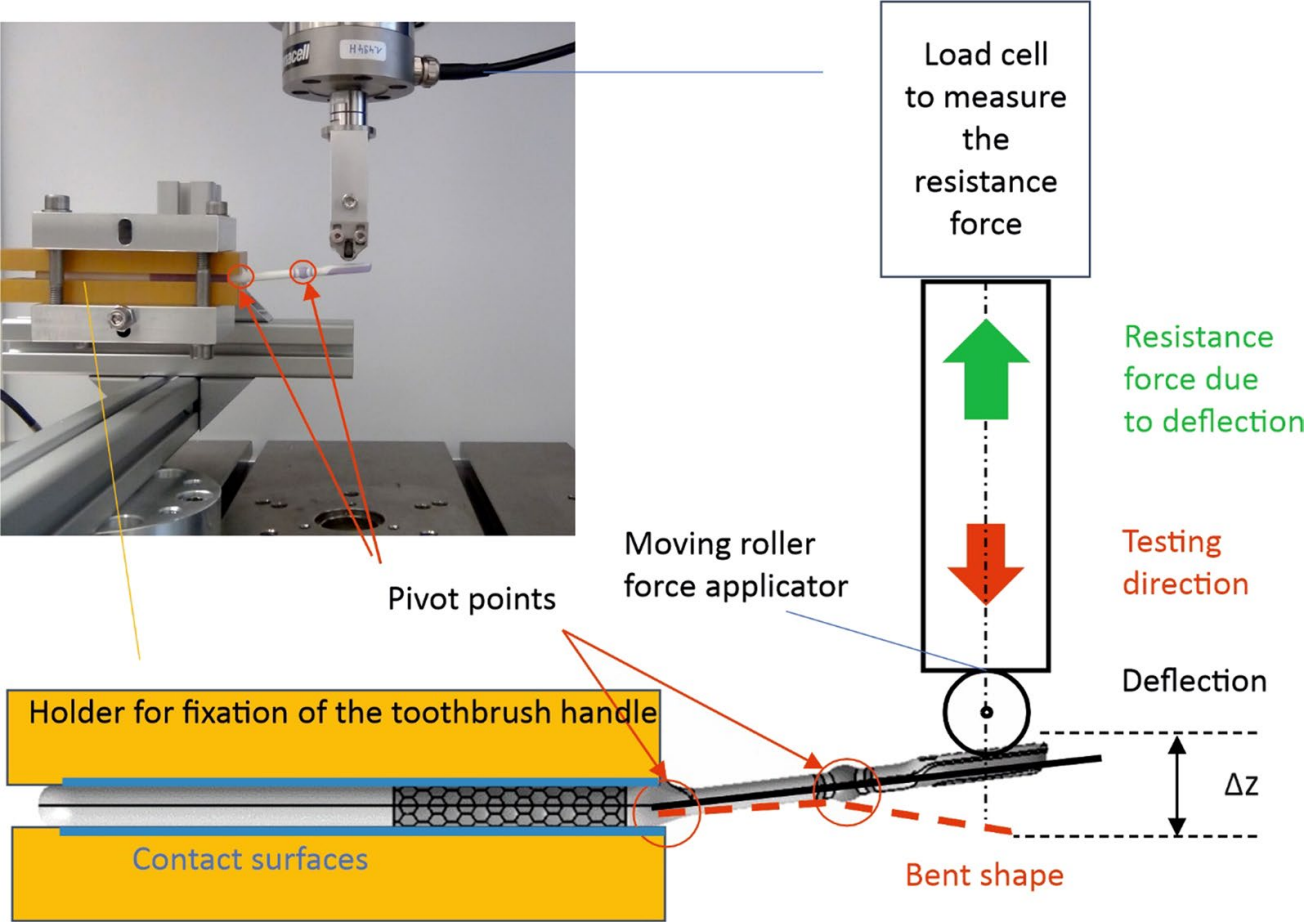
Schematic for mechanical testing of toothbrush samples. The toothbrush handles were fixed in position using a 3D-printed form-fitted holder and epoxy resin to prevent uncontrolled bending of the toothbrush. The form-fitting holder was necessary because a conventional fixation for the toothbrushes creates additional pivot points. This would result in bending of the handle while testing, which does not reflect how toothbrushes behave in practical use, and affects the resulting forces. Using individual sample holders increases the contact surface on the handle and reduces the amount of pivot points. This provides a good representation of actual toothbrushing and minimises the influence of the handle on the measurements

This study compared test toothbrushes (flexible neck, ten samples without bristles) with the control toothbrush (rigid neck, ten samples without bristles, Jubilee Toothbrush). A universal testing device (ElectroPuls™ E10000 Linear-Torsion All-Electric Dynamic Test Instrument by Instron, GmbH, Germany) was used to measure the resistance force of the toothbrush heads while deflecting them with a roller force applicator equipped with a load cell (Dynacell 250 N by Instron, GmbH, Germany) (Fig. 1).

**Fig. 2:**
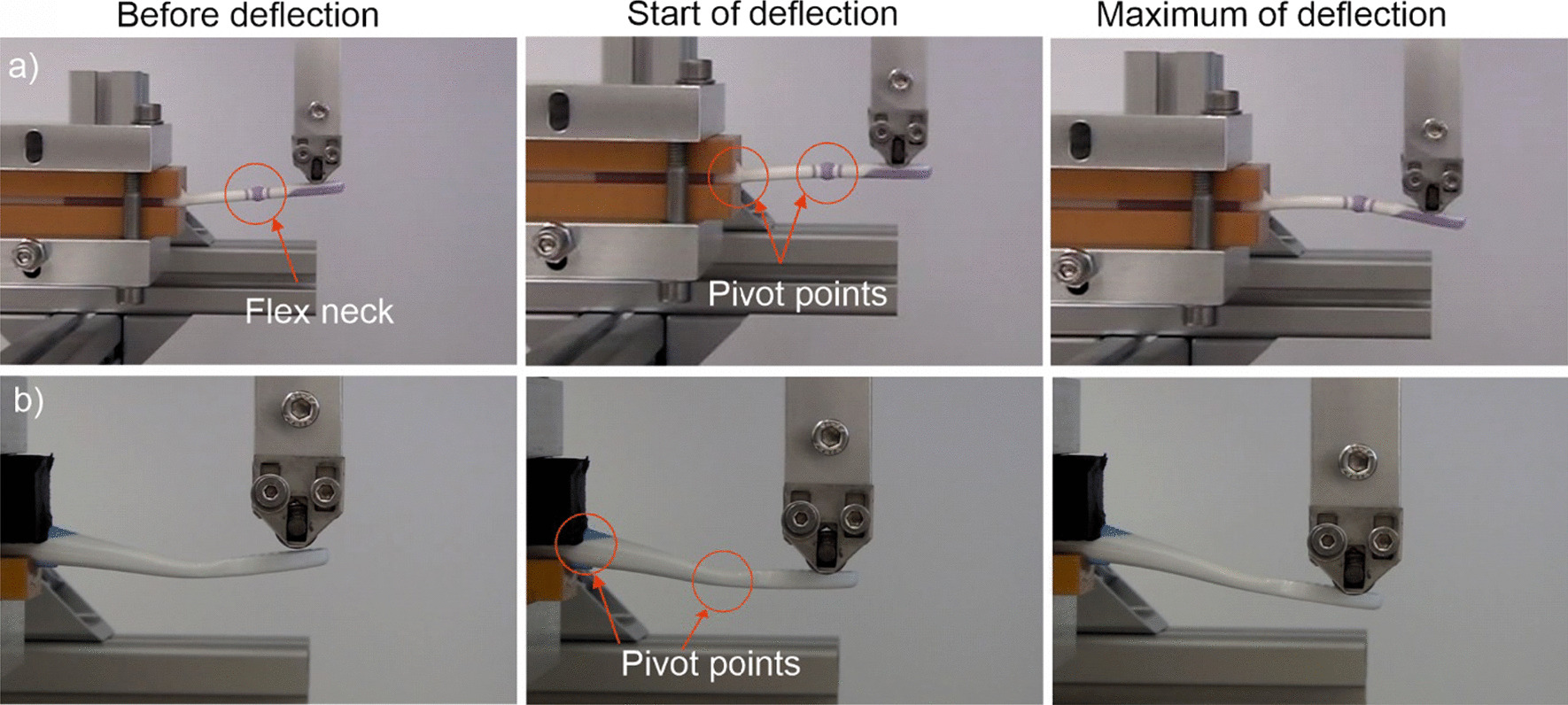
Mechanical testing of the test (**a**) and control toothbrush (**b**)

To record force-deflection diagrams, ten samples per toothbrush type were tested. Force was applied to the handle until a maximum head deflection of 10 mm was reached. In the deflection test, the starting position of the roller force applicator was 1–2 mm above the toothbrush head and 60 mm away from the thumb position of the handle. For testing, the roller force applicator moved downwards (10 mm) and deflected the toothbrush head. The resulting resistance force was measured by a load cell (Figs. 1, 2a and 2b).

### Study 2—Cleaning efficacy by plaque planimetry

**Fig. 3:**
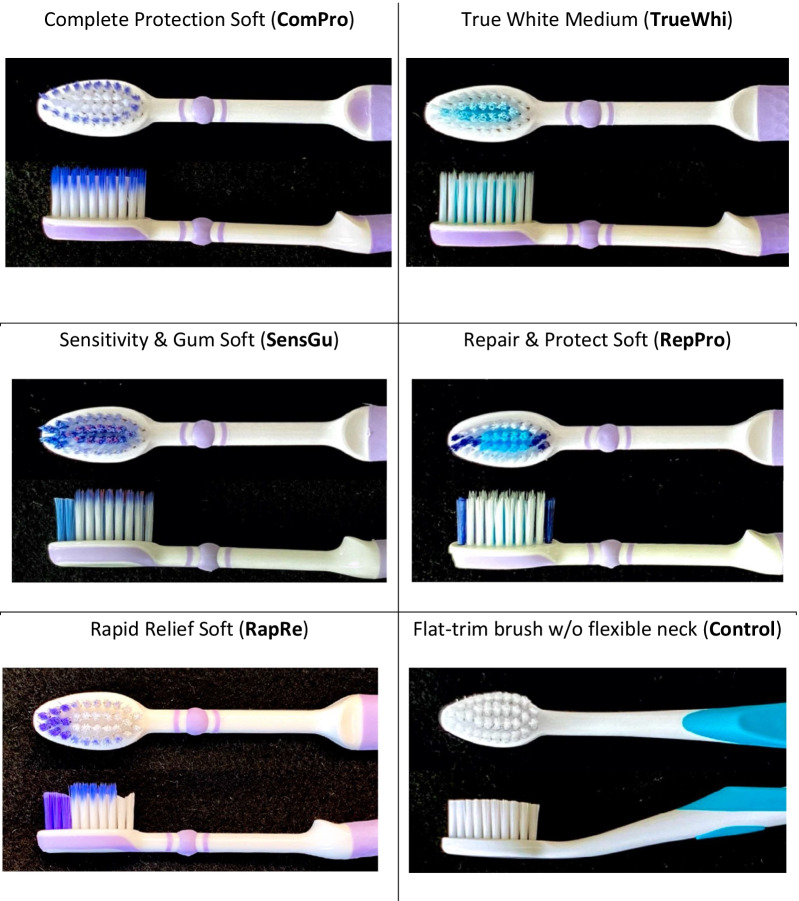
Test and control toothbrushes

The pre-test was conducted to compare brushing at forces of 2.5 N and 3.5 N. This showed no significant differences in toothbrush performance between those two forces. It was therefore decided to test all six toothbrushes with the lower force of 2.5 N only. This study used an in vitro robot test of six toothbrushes on typodonts in anatomic position (KaVo®) assessing efficacy with automated plaque planimetry (APP) assessments at 30 fields per tooth (Additional File 1: Fig. S1). The five test versus one control toothbrushes assessed in this study can be seen in Fig. 3. The test toothbrushes were: Complete Protection Soft (ComPro), True White Medium (TrueWhi), Sensitivity & Gum Soft (SensGu), Repair and Protect Soft (RepPro) and Rapid Relief Soft (RapRe). The control or reference toothbrush was the Jubilee Toothbrush (RefJub).

Three different brushing movements were assessed in Study 2: horizontal, vertical and rotating. The brushing force was 2.5 N and seven distinct runs were conducted per movement. The teeth were tested at sites buccally and lingually, mesially and distally with full statistical support tests.

The evaluation process compared five test toothbrushes with the control toothbrush. This involved differentiation of gumline and interproximal regions (ABCDF-fields) of buccal and lingual sites (the locations of these APP tooth fields are given in the Supplementary Material). Evaluation also included cleaning efficacy tooth-by-tooth of all interproximal, buccal and lingual sites; root fields W buccally and W lingually, W1W2 mesially and W1W2 distally; total cleaning efficacy of all buccal and lingual, mesial and distal, coronal and root sites (30 fields per tooth), and illustration of brushing efficacy at these sites with box plots, error bars and tables (see Additional File 1: Fig. S2 for locations of these tooth fields). In the statistical analysis, the Kolmogorov–Smirnov test was applied to test the cleaning efficacy of the toothbrushes by tooth surface variables. However, the null hypothesis of normality was rejected as performance of the toothbrushes was not normally distributed with equivalent variances, therefore analysis was performed using the non-parametric Wilcoxon–Mann–Whitney (WMW) test. All toothbrushes were tested against each other. The WMW test can be applied to ordinal or unknown disturbances, contrary to the t-test, and it is nearly as efficient as the parametric t-test (power efficiency of the WMW test: 95% > power efficiency > 90%).

For all two-tailed tests of differences in cleaning efficacy between the six toothbrushes, the significance level was set at the basic p-value of α = 0.05 (5%). Statistical analyses were performed using IBM SPSS® professional statistics software, release 26, 64-bit version.

## Results

### Study 1—Mechanical testing of toothbrush deflection 

**Fig. 4:**
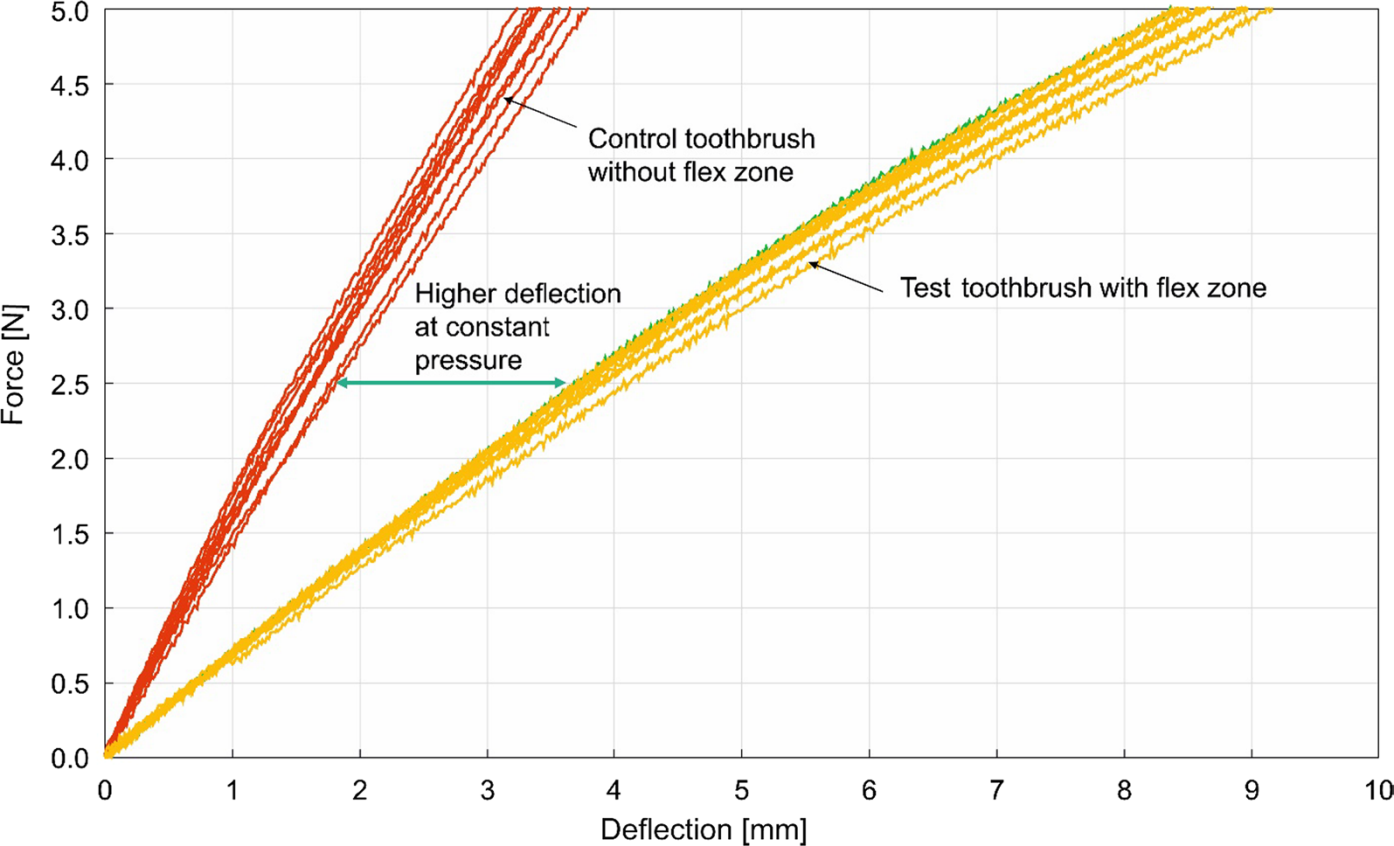
Force–deflection curves for test vs control toothbrushes. At the same force, the test toothbrush is deflected 2–2.5 times more than the control toothbrush

During mechanical testing, the test and the control toothbrush neck handles behaved differently. For the test toothbrushes with flexible necks, at the starting position, the head of the toothbrush was straight, although following the start of deflection, the brush head flexed at both pivot points (thumb position and flexible neck, see Fig. 2a/2b). This flexing was slightly larger at the flexible neck. At maximum deflection, the brush head was significantly bent. The bending occurred mainly through deformation at the flexible neck. For the control toothbrush, at the starting position, the head of the toothbrush was unchanged with a small angle at the end of the head. After the deflection started, the toothbrush head was bent at both pivot points (thumb position and start of head). The bending was slightly larger at the toothbrush head. At maximum deflection, the toothbrush head was significantly bent due to deformation of the smallest cross section at the end of the toothbrush head.When force was further exerted on a test toothbrush, the bending occurred mainly at the flex zone, whereas the entire neck and head were deflected for the control toothbrush. At an applied force of 2.5 N, a mean deflection of 3.9 ± 0.1 mm was measured for the test toothbrush, and 1.6 ± 0.1 mm for the control toothbrush (Fig. 4). The entire force-deflection progression showed that the test toothbrush is deflected 2–2.5 times more than the control toothbrush when the same bending force was applied (Fig. 4).

### Study 2—Cleaning efficacy by plaque planimetry

After a pre-test assessment of two different brushing forces (2.5 N vs 3.5 N) it was decided to test all the toothbrushes at the lower brushing force of 2.5 N only, which a bending assessment indicated was associated with 75% bending of the test toothbrushes. All of the five test toothbrushes were statistically superior in performance in terms of artificial plaque removal (%) compared to the control toothbrush (*p* < 0.001 for most comparisons). The superior in vitro performance of the test toothbrushes was maintained at all crown risk areas, including ABCDF next to the gumline, mesial and distal inbetween fields. This is shown in the WMW U statistics and associated probabilities for total cleaning effects of the five test toothbrushes versus the control toothbrush in Table 1 and in Additional File 1: Tables 4–6.

**Table 1:** Cleaning efficacy using A. horizontal, B. rotating and C. vertical toothbrush movements

A. Horizontal brushing movement					
Toothbrush type Mean total % plaque removal ± SD	**ComPro** 69.4 ± 2.5	**TrueWhi** 63.7 ± 4.1	**SensGu** 65.9 ± 2.1	**RepPro** 64.6 ± 3.0	**RapRe** 65.0 ± 1.8
ComPro 69.4 ± 2.5	**N/A**	–	–	–	–
TrueWhi 63.7 ± 4.1	4.0 *p* = 0.007*	**N/A**	–	–	–
SensGu 65.9 ± 2.1	5.0 *p* = 0.011*	NS	**N/A**	–	–
RepPro 64.6 ± 3.0	4.0 *p* = 0.007*	6.0 *p* = 0.035*	3.0 *p* = 0.004*	**N/A**	–
RapRe 65.0 ± 1.8	3.0 *p* = 0.004*	NS	NS	NS	**N/A**
Control 47.5 ± 2.4	0.0 *p* = 0.001	0.0 *p* = 0.001	0.0 *p* = 0.001	0.0 *p* = 0.001	0.0 *p* = 0.001

B. Rotating brushing movement					
Toothbrush type Mean total % plaque removal ± SD	**ComPro** 69.8 ± 3.6	**TrueWhi** 64.6 ± 1.6	**SensGu** 66.0 ± 2.7	**RepPro** 63.3 ± 5.2	**RapRe** 65.6 ± 2.6
ComPro 69.8 ± 3.6	N/A	–	–	–	–
TrueWhi 64.6 ± 1.6	5.0 *p* = 0.011*	N/A	–	–	–
SensGu 66.0 ± 2.7	8.0 *p* = 0.038*	NS	N/A	–	–
RepPro 63.3 ± 5.2	8.0 *p* = 0.038*	NS	NS	N/A	–
RapRe 65.6 ± 2.6	8.0 *p* = 0.038*	NS	NS	NS	N/A
Control 46.0 ± 2.2	0.0 *p* = 0.001	0.0 *p* = 0.001	0.0 *p* = 0.001	0.0 *p* = 0.001	0.0 *p* = 0.001

C. Vertical brushing movement					
Toothbrush type Mean total % plaque removal ± SD	**ComPro** 53.2 ± 6.1	**TrueWhi** 36.5 ± 5.0	**SensGu** 40.2 ± 5.7	**RepPro** 51.5 ± 5.3	**RapRe** 51.1 ± 2.1
ComPro 53.2 ± 6.1	N/A	–	–	–	–
TrueWhi 36.5 ± 5.0	2.0 *p* = 0.002	N/A	–	–	–
SensGu 40.2 ± 5.7	4.0 *p* = 0.007*	NS	N/A	–	–
RepPro 51.5 ± 5.3	NS	1.0 *p* = 0.001	4.0 *p* = 0.007*	N/A	–
RapRe 51.1 ± 2.1	NS	0.0 *p* = 0.001	0.0 *p* = 0.001	24.0 *p* = 1.000	N/A
Control 35.9 ± 2.0	0.0 *p* = 0.001	NS	NS	0.0 *p* = 0.001	0.0 *p* = 0.001

Data are Wilcoxon–Mann–Whitney (WMW) U-statistic and *p*-values. The cleaning efficacy (% plaque removal) of six toothbrushes (ComPro, TrueWhi, SensGu, RepPro, RapRe, Control) was assessed using APP-index at 30 fields per tooth using three different brushing movements. The total values given are derived from % plaque removal using brushing of the following areas: buccal, mesial, distal, ABCDF buccal, ABCDF lingual, W buccal, W lingual, W1 + W2 mesial, W1 + W2 distal. The cleaning efficacy (% plaque removal) of each single toothbrush was compared against each other via a WMW test (*not significant using Bonferroni correction). APP: automated plaque planimetry; ComPro: Complete Protection Soft; N/A: not applicable; NS: not significant (on either test); RapRe: Rapid Relief Soft; RepPro: Repair & Protect Soft; SD, standard deviation; SensGu: Sensitivity & Gum Soft; TrueWhi: True White Medium; WMW: Wilcoxon–Mann–Whitney.

**Fig. 5:**
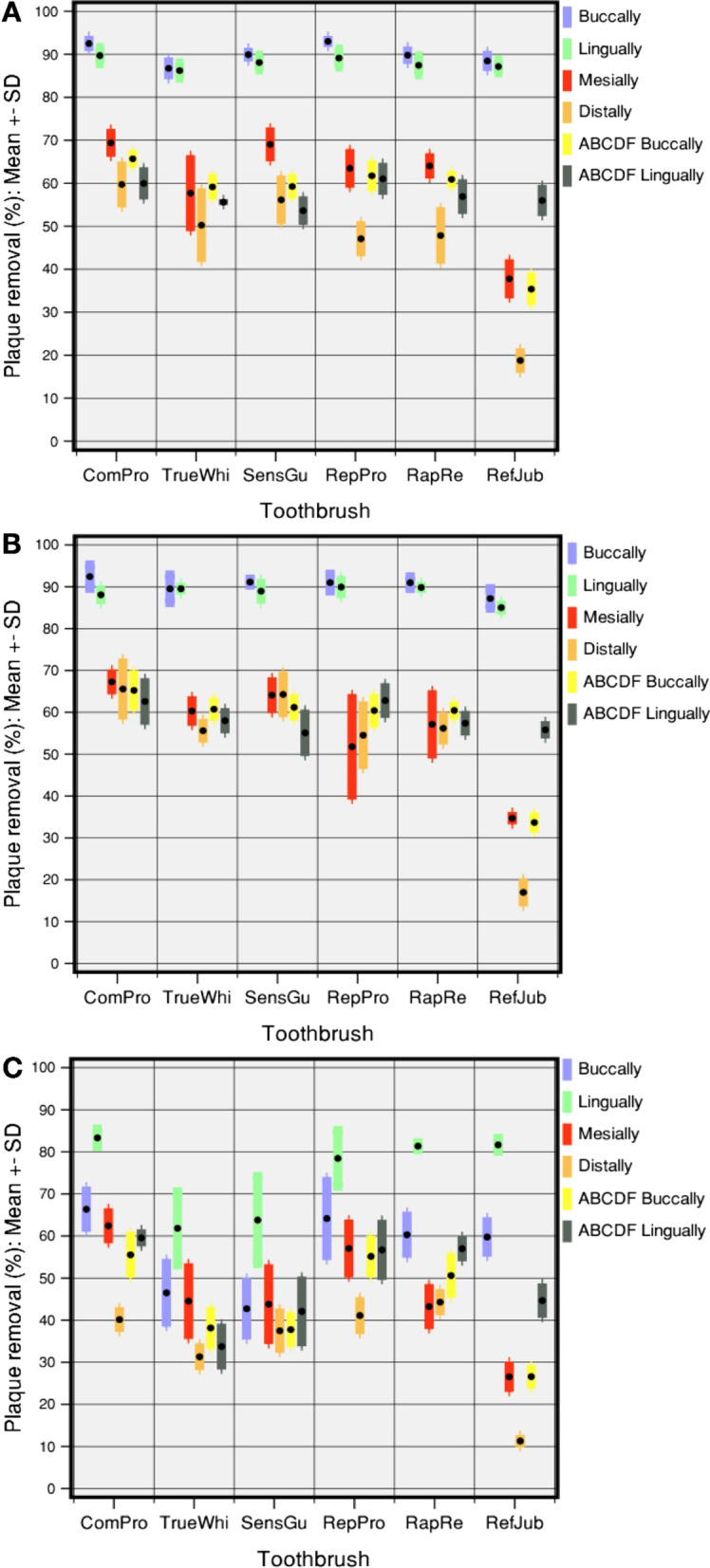
Cleaning efficacy across planimetrical coronal fields using A. horizontal, B. rotating and C. vertical brushing. Plaque removal percentage at the four sites per each tooth (smooth surfaces buccally and lingually, in between teeth mesially and distally, ABCDF risk area next to the gumline). Black dots represent mean values, coloured error bars represent + / − 1 standard deviation

A comparison of interdental and gumline cleaning for the test toothbrushes versus the control toothbrush is given in Fig. 5. The plots show means and standard deviations (as error bars) for plaque removal by the six toothbrushes using horizontal, rotating and vertical brushing movements. In each case, the superiority in performance by the flexible-neck designs compared to the control toothbrush was shown. This difference was most apparent for mesial, distal and ABCDF buccal locations for all three types of brushing action.

For horizontal, rotating and vertical cleaning efficacy parameters, the ComPro toothbrush showed the best overall plaque removal performance. The next best-performing test toothbrushes were the RepPro and the RapRe. These three toothbrushes tended to perform better when compared to the other test toothbrush counterparts (TrueWhi and SensGu) but the differences between them were quite small (Fig. 5, Table 1, and Supplementary Fig. 3). However, all of the test toothbrushes performed significantly better than the rigid control toothbrush, which was the poorest-performing toothbrush (Fig. 5, Table 1, and Additional File 1: Fig. 3).

The average cleaning score across three movements for the ComPro test toothbrush was 64.12% compared to the control toothbrush, which was 43.11%. The in vitro brushing efficacy was generally greater for the five test toothbrushes in terms of access to interdental spaces, exposed root surfaces and differing tooth types, compared to the control toothbrush. Horizontal and rotating brush movements with the test toothbrushes were significantly more effective in removing simulated plaque than for the control toothbrush. Vertical brushing movements were also more effective with the test toothbrushes, although only for 3 out of 5 test toothbrushes.

An analysis of single replicated human teeth showed that there was optimum plaque control at incisors (up to 99.75%) after horizontal brushing in interdental risk areas, followed by wisdom teeth (up to 64.06%) then canines, premolars and molars (up to 45.72%) (See Additional File 1: Tables 1–6). 

## Discussion

The mechanical testing in the two reported studies shows that the addition of a flexible neck to the toothbrush designs significantly reduces handle stiffness in the neck and head regions compared to rigid-neck toothbrushes. This could help reduce the force applied to the oral mucosa and tooth surfaces. Moreover, this flexibility is apparent at force levels typically used in manual toothbrushing. The tests of toothbrush deflection clearly showed very different force-deflection curves, with the test toothbrush showing 2.5 times lower measured force at the head than the control toothbrush. This deflection arose as a result of the flexible neck component rather than different leverage effects or the angle of the brush heads. The presence of the flexible neck results in smaller moment arm for the test toothbrushes, resulting in lower bending moments on the tooth surface compared to the control toothbrushes. The lower force and moment produced by the test toothbrushes can help provide a more gentle brushing action and could therefore help prevent overbrushing and gum damage. 

The force-deflection curve indicates that when used for cleaning, the test toothbrushes could theorectically have a more controlled force distribution of brushes on the teeth. The curve also indicates that high forces are achieved with small deformations. The force steps are smaller with the test compared to the control toothbrush, enabling improved force control and better cleaning.

The comparison of the five flexible-neck test designs with the control design consistently demonstrated improved in vitro performance in terms of cleaning efficacy over multiple different tooth surfaces when the brushing movement was either horizontal or rotating. With vertical brushing movement, however, the clear superiority of performance was less marked. This is due to the fact that how the flexible neck bends supports horizontal/rotating motion, but vertical motion limits the force transfer, and the neck handle reacts in a similar manner to a rigid toothbrush. It should be noted that the most common brushing actions are horizontal and rotating [13, 24, 25] and their efficacy is clearly supported by the current findings, even though the efficacy achieved by vertical brushing movements was at least comparable to that achieved by rigid brushing handles. 

In this in vitro model, when compared to the rigid-necked control toothbrush, the flexible toothbrush neck appeared to contribute to cleaning in hidden biofilm-accumulating regions inbetween the teeth and around exposed roots. The majority of consumers worldwide, when using toothbrushes, exhibit random brushing habits with horizontal scrubbing, rotating and gliding actions. Whilst our robot model is able to replicate highly standardised human brushing techniques [13], the model is unable to reflect sensory feedback affecting individual random clinical brushing technique and dynamic force adaptation. Our data suggest that toothbrushes with high neck flexibility could be suited to typical human techniques than more rigid designs (Additional Files 2,3,4: Videos 1–3). To appropiately investigate if toothbrushes with greater neck flexibility confer improved cleaning efficacy in people, it will need to be investigated in clinical trials. 

An alternative strategy to reduce the force transmitted to the teeth during brushing is to use softer bristles, and previous studies have investigated the relationship between bristle stiffness and abrasive dentine wear, a correlate of transmitted force [23, 26–30]. However, whilst some studies suggest that softer bristles are associated reduced dentine abrasion [23, 27, 28], others indicate the opposite [26, 31]. Other factors in addition to bristle stiffness, such as their density, diameter, length, orientation and arrangement, as well as toothpaste properties are also thought to influence the findings of these studies [29, 30]. As bristle configuration were not standardised across the test brushes, these factors could have influenced the results in this study, and how all of these factors interact with toothbrush neck flexibility are interesting topics for further study.

The analysis of single teeth also showed superior cleaning performance at multiple different sites by the flexible-neck designs. This cleaning was most effective for incisors, followed by wisdom teeth, canines, premolars and molars. This could be an issue of access to individual teeth and may be worth further investigation. The cleaning efficacy study advances previous work on robot typodonts, real toothbrushing, and visualisation and quantification of simulated plaque removal [13–16]. This technology is important for the accurate assessment of tooth cleaning potential and in the development of new approaches in oral hygiene. 

## Conclusions

These studies confirm that the novel toothbrush designs, with the addition of a flexible component in the neck of the test toothbrushes, increase neck flexibility under typical toothbrushing forces. This design may reduce the force transmitted from the toothbrush to dental surfaces, resulting in lower shear and compressive stresses, and consequently potentially less damage to the teeth and gums. 

The cleaning efficacy of the test toothbrushes, as tested by using an in vitro robot model employing three different brushing movements and biophysically standardised brushing forces (2.5 N), indicates that the five test flexible-neck designs provide comparatively improved simulated plaque control at all risk areas and their single planimetrical fields, when compared to their rigid-neck counterpart. Theoretically this couldcontribute to the prevention of dental caries and gingivitis, and potentially improve dental health. However, it would need to be investigated in subsequent clinical trials.

In future planned studies, torsional movements around the long axis of the toothbrush handle, and their effects, could also be measured. In addition, the inner stress distribution of the entire toothbrush body could be determined to better understand mechanical behaviour during force exertion. Experiments that investigate how different bristle heads interact with flexible neck designs to reduce the force transmitted during brushing would also be of potential interest. These studies could provide further insights, leading to improvements in toothbrush design and performance for better oral health.

## Supplementary Information

**Additional file 1.** Clinically validated robot testing of manual toothbrushes with three brushing movements and brushing force of 2.5 N, planimetrical fields around tooth crowns and roots at four incisors, canines, two premolars and three molars. Plaque assessment at four sites per tooth in risk areas interdentally, next to gum line and at exposed root surfaces. Plaque removal efficacy demonstrated with error bars and in tables, summarizing the statistical outcome for all six toothbrushes, three brushing movements and eight risk areas per tooth (next to gum line buccally and lingually, proximal mesially and distally, exposed root surfaces buccally and lingually, and mesially and distally).

**Additional file 2.** Video sequence of vertical brushing movement, brushing force 2.5 N, toothbrush Complete Protection Soft.

**Additional file 3.** Video sequence of horizontal brushing movement, brushing force 2.5 N, toothbrush Complete Protection Soft.

**Additional file 4.** Video sequence of rotating brushing movement, brushing force 2.5 N, toothbrush Complete Protection Soft.

## Data Availability

The complete datasets generated and/or analysed during the current study are not publicly available in the manuscript due to their lack of relevance to this article and word count considerations. Although, they are available from the corresponding author on reasonable request.

## Abbreviations

3D: Three dimensional
APP: Automated plaque planimetry
ComPro: Complete Protection Soft
N: Newton
RapRe: Rapid Relief Soft
RefJub: Jubilee Toothbrush
RepPro: Repair and Protect Soft
SensGu: Sensitivity and Gum Soft
TrueWhi: True White Medium
WMW: Wilcoxon–Mann–Whitney
