# Supplementary material for: Robot and mechanical testing of a specialist manual toothbrush for cleaning efficacy and improved force control
Source: BMC Oral Health. 2022 Jun 8;22:225. doi: 10.1186/s12903-022-02211-4 (PMC9175444; doi:10.1186/s12903-022-02211-4)
Supplement: Supplementary file 1 — Additional file 1. Clinically validated robot testing of manual toothbrushes with three brushing movements and brushing force of 2.5 N, planimetrical fields around tooth crowns and roots at four incisors, canines, two premolars and three molars. Plaque assessment at four sites per tooth in risk areas interdentally, next to gum line and at exposed root surfaces. Plaque removal efficacy demonstrated with error bars and in tables, summarizing the statistical outcome for all six toothbrushes, three brushing movements and eight risk areas per tooth (next to gum line buccally and lingually, proximal mesially and distally, exposed root surfaces buccally and lingually, and mesially and distally). [file 12903_2022_2211_MOESM1_ESM.docx]

**Additional file 1.** Clinically validated robot testing of manual toothbrushes with three brushing movements and brushing force of 2.5 N, planimetrical fields around tooth crowns and roots at four incisors, canines, two premolars and three molars. Plaque assessment at four sites per tooth in risk areas interdentally, next to gum line and at exposed root surfaces. Plaque removal efficacy demonstrated with error bars and in tables, summarizing the statistical outcome for all six toothbrushes, three brushing movements and eight risk areas per tooth (next to gum line buccally and lingually, proximal mesially and distally, exposed root surfaces buccally and lingually, and mesially and distally).

**Figure S1.** Clinically validated ORMED brushing robot with replicated human teeth and brushing force assessment

**
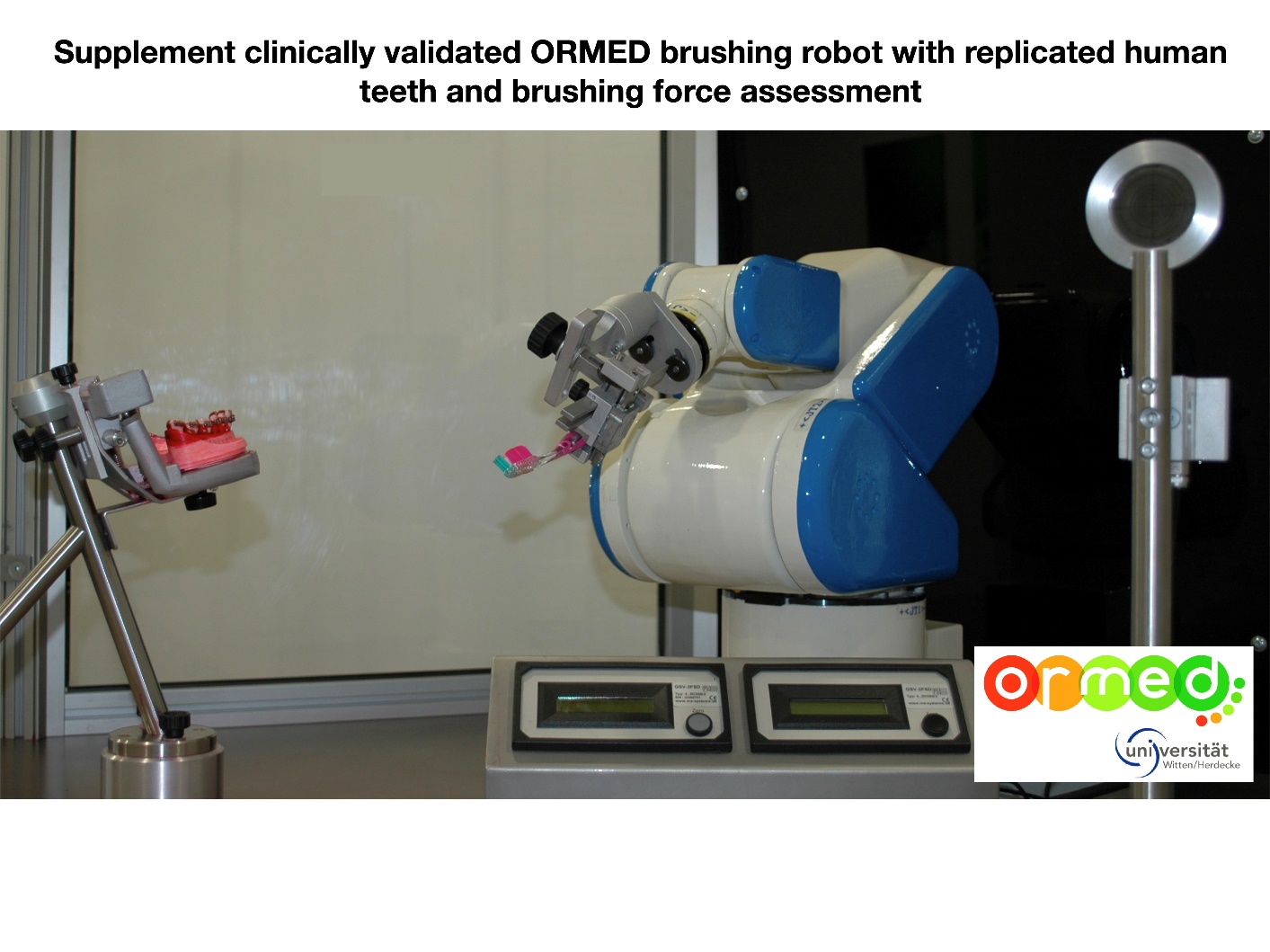
**

**Figure S2.** Automated plaque planimetry index (APP-Index) tooth locations: A. left: APP index buccally (towards the cheek); right APP Index lingually (towards the tongue); B. APP index mesially (proximal in-between teeth, anterior side, right: APP-Index distally (proximal in-between teeth, posterior side)

**
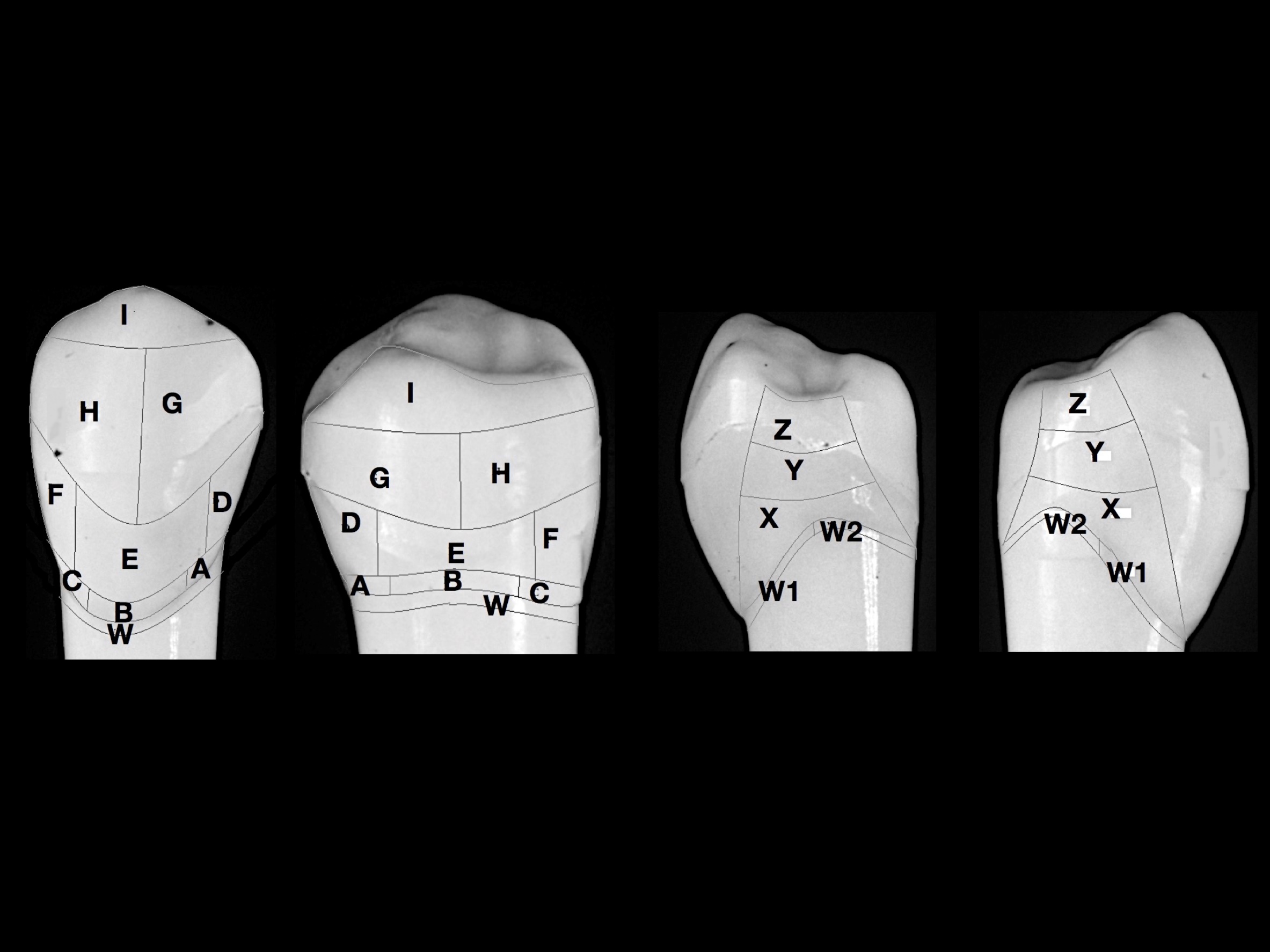
**

**A.**

**
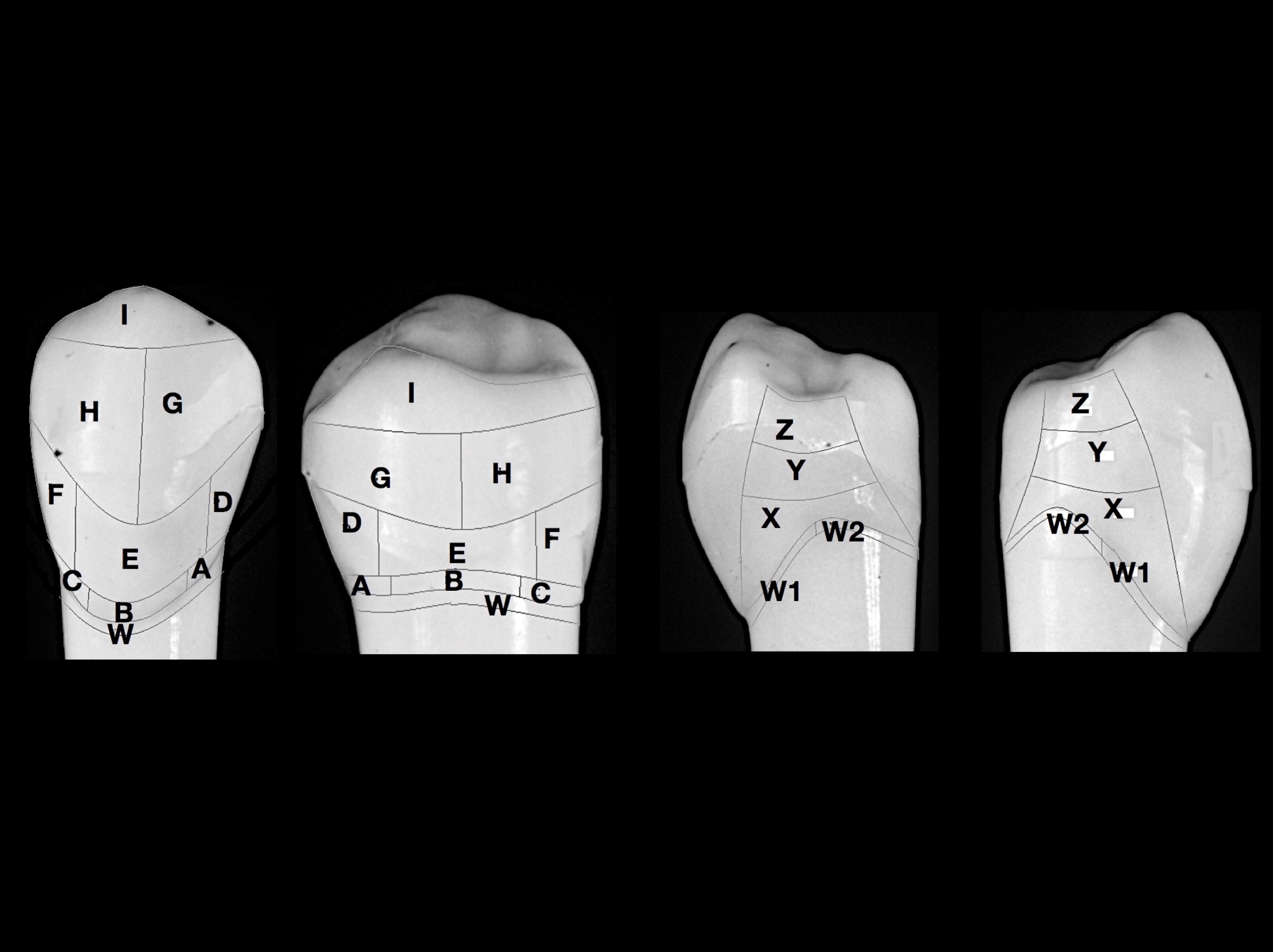
**

**B.**

**Figure S3.** Cleaning efficacy across root sites using A. horizontal, B. rotating and C. vertical brushing.

**
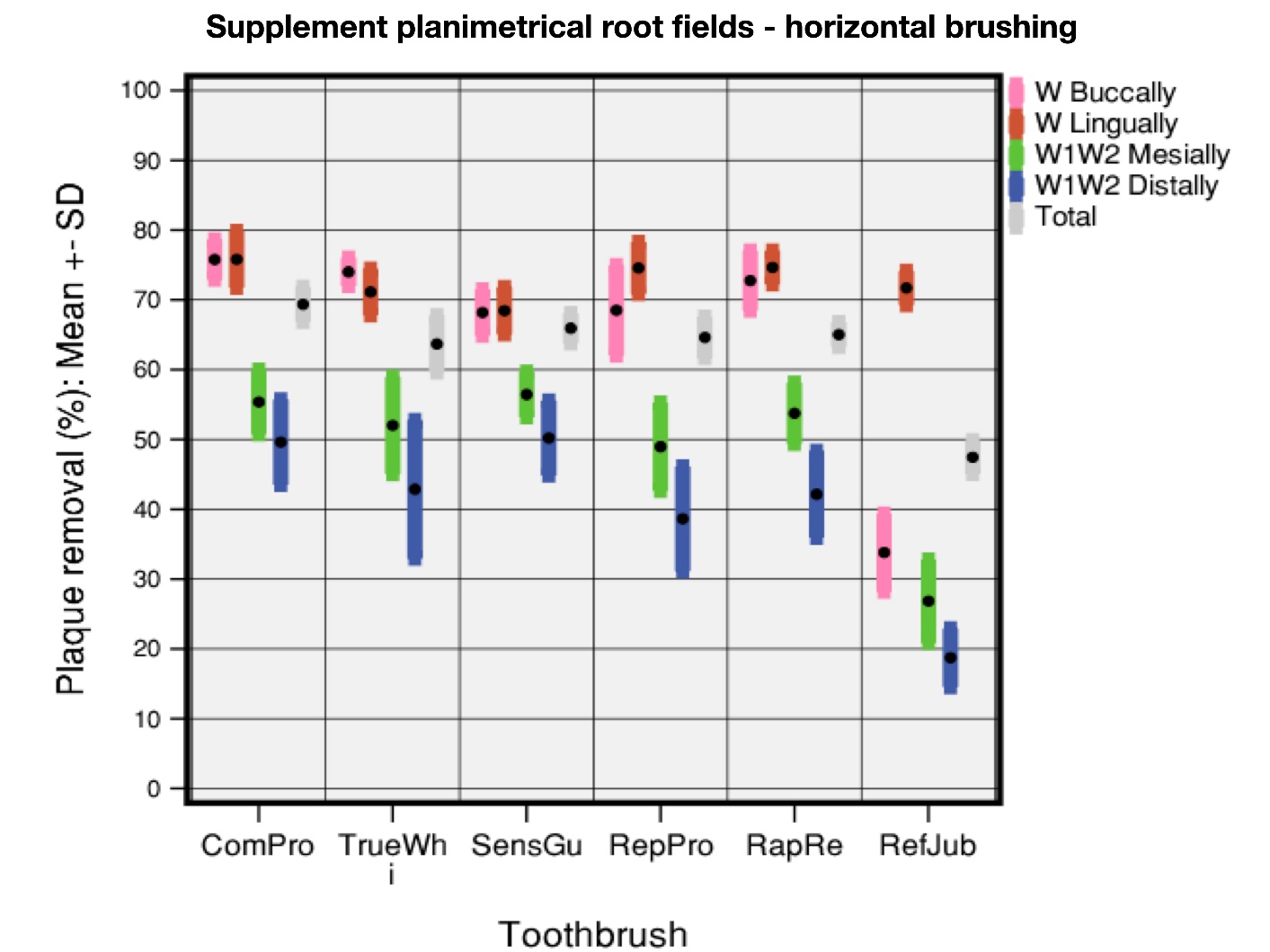
**

1. **Horizontal brushing**

**
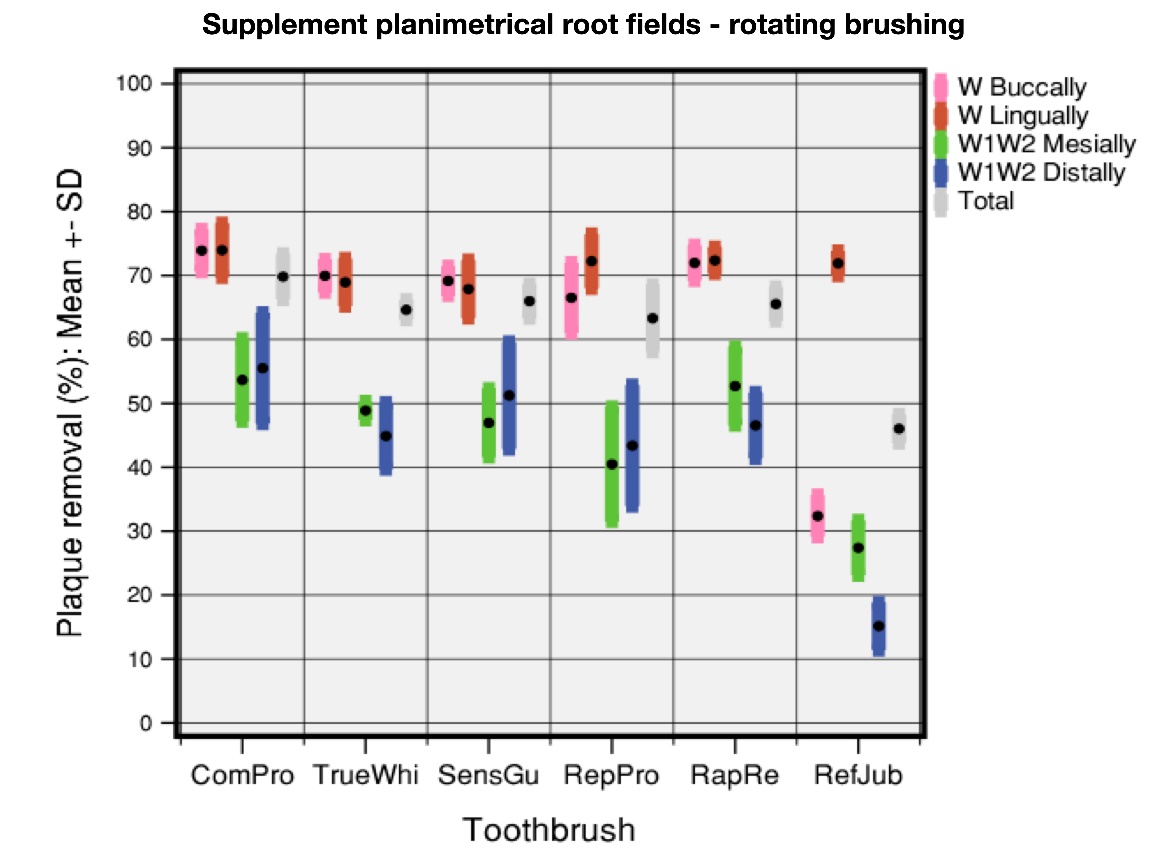
**

1. **Rotating brushing**

**
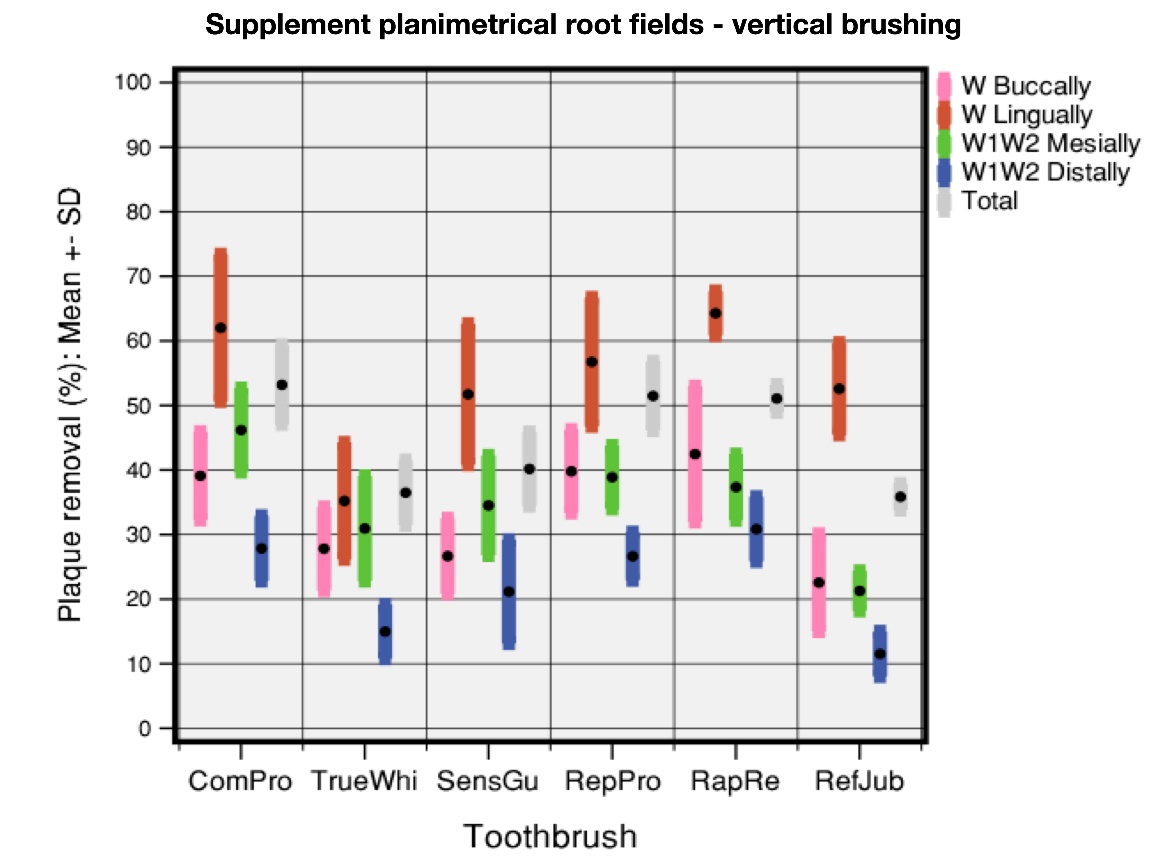
**

1. **Vertical brushing**

**Table S1** Means (M)) and standard deviations (SD), medians (Med) and interquartile ranges (IQR; LL = lower limit, UL = upper limit) of cleaning efficacy of the 5 test toothbrushes und 1 control toothbrush: **Horizontal brushing movement**

**Table S1 (continued)** Means (M)) and standard deviations (SD), medians (Med) and interquartile ranges (IQR; LL = lower limit, UL = upper limit) of cleaning efficacy: **Horizontal brushing movement**

**Table S1 (continued)** Means (M)) and standard deviations (SD), medians (Med) and interquartile ranges (IQR; LL = lower limit, UL = upper limit) of cleaning efficacy: **Horizontal brushing movement**

**Table S2** Means (M)) and standard deviations (SD), medians (Med) and interquartile ranges (IQR; LL = lower limit, UL = upper limit) of cleaning efficacy: **Rotating brushing movement**

**Table S2 (continued)** Means (M)) and standard deviations (SD), medians (Med) and interquartile ranges (IQR; LL = lower limit, UL = upper limit) of cleaning efficacy: **Rotating brushing movement**

**Table S2 (continued)** Means (M)) and standard deviations (SD), medians (Med) and interquartile ranges (IQR; LL = lower limit, UL = upper limit) of cleaning efficacy: **Rotating brushing movement**

**Table S3** (Means (M)) and standard deviations (SD), medians (Med) and interquartile ranges (IQR; LL = lower limit, UL = upper limit) of cleaning efficacy: **Vertical brushing movement**

**Table S3 (continued)** (Means (M)) and standard deviations (SD), medians (Med) and interquartile ranges (IQR; LL = lower limit, UL = upper limit) of cleaning efficacy: **Vertical brushing movement**

**Table S3 (continued)** (Means (M)) and standard deviations (SD), medians (Med) and interquartile ranges (IQR; LL = lower limit, UL = upper limit) of cleaning efficacy: **Vertical brushing movement**

**Table S4** Mann-Whitney-Test of cleaning efficacy (% plaque removal): contrasts of the six toothbrushes – **Horizontal brushing movement**

**Table S4 (continued)** Mann-Whitney-Test of cleaning efficacy (% plaque removal): contrasts of the six toothbrushes **- Horizontal brushing movement**

**Table S4 (continued)** Mann-Whitney-Test of cleaning efficacy (%plaque removal): contrasts of the six toothbrushes **- Horizontal brushing movement**

**Table S4 (continued)** Mann-Whitney-Test of cleaning efficacy (%plaque removal): contrasts of the six toothbrushes **- Horizontal brushing movement**

**Table S4 (continued)** Mann-Whitney-Test of cleaning efficacy (%plaque removal): contrasts of the six toothbrushes **- Horizontal brushing movement**

U = test statistic of the Mann-Whitney-Test

Z = normalized test statistic of the Mann-Whitney-Test

p = significance value

*significant (p≤0.05)

**very significant (p≤0.01)

***highly significant (p≤0.001)

Yellow marking = not significant using Bonferroni correction

**Table S5** Mann-Whitney-Test of cleaning efficacy (%plaque removal): contrasts of the six toothbrushes **- Rotating brushing movement**

**Table S5 (continued).** Mann-Whitney-Test of cleaning efficacy (%plaque removal): contrasts of the six toothbrushes **- Rotating brushing movement**

**Table S5 (continued).** Mann-Whitney-Test of cleaning efficacy (%plaque removal): contrasts of the six toothbrushes **- Rotating brushing movement**

**Table S5 (continued).** Mann-Whitney-Test of cleaning efficacy (%plaque removal): contrasts of the six toothbrushes **- Rotating brushing movement**

**Table S5 (continued).** Mann-Whitney-Test of cleaning efficacy (%plaque removal): contrasts of the six toothbrushes **- Rotating brushing movement**

U = test statistic of the Mann-Whitney-Test

Z = normalized test statistic of the Mann-Whitney-Test

p = significance value

*significant (p≤0.05)

**very significant (p≤0.01)

***highly significant (p≤0.001)

Yellow marking = not significant using Bonferroni correction

**Table S6.** Mann-Whitney-Test of cleaning efficacy (%plaque removal): contrasts of the six toothbrushes **- Vertical brushing movement**

**Table S6 (continued).** Mann-Whitney-Test of cleaning efficacy (%plaque removal): contrasts of the six toothbrushes **- Vertical brushing movement**

**Table S6 (continued).** Mann-Whitney-Test of cleaning efficacy (%plaque removal): contrasts of the six toothbrushes **- Vertical brushing movement**

**Table S6 (continued).** Mann-Whitney-Test of cleaning efficacy (%plaque removal): contrasts of the six toothbrushes **- Vertical brushing movement**

**Table S6 (continued).** Mann-Whitney-Test of cleaning efficacy (%plaque removal): contrasts of the six toothbrushes **- Vertical brushing movement**

U = test statistic of the Mann-Whitney-Test

Z = normalized test statistic of the Mann-Whitney-Test

p = significance value

*significant (p≤0.05)

**very significant (p≤0.01)

***highly significant (p≤0.001)

Yellow marking = not significant using Bonferroni correction
